# Supplementary material for: Expression of FIBCD1 by intestinal epithelial cells alleviates inflammation-driven tumorigenesis in a mouse model of colorectal cancer
Source: Front Oncol. 2023 Nov 28;13:1280891. doi: 10.3389/fonc.2023.1280891 (PMC10715588; doi:10.3389/fonc.2023.1280891)
Supplement: Supplementary file 1 [file DataSheet_1.pdf]

Supplementary Material

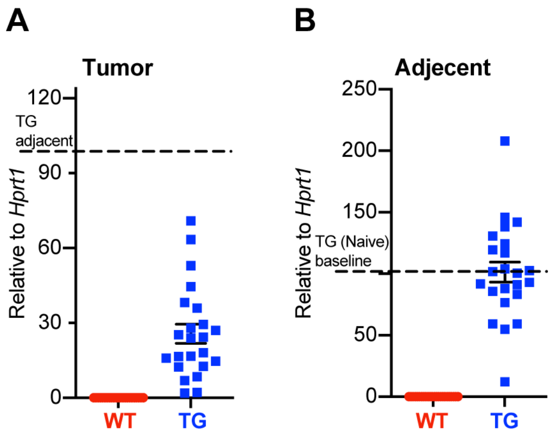

Supplementary Figure 1. FIBCD1 expression in tissues from WT and transgenic littermates. (A) Relative expression of *Fibcd1* in tumor tissues isolated from WT and TG littermates (n=23-24 mice per group). The dotted line indicates the average expression level in adjacent non-tumorigenic colonic tissues isolated from TG littermate controls. **(B)** Relative expression of *Fibcd1* in non-tumorigenic adjacent tissues isolated from WT and TG littermates (n=23-24 mice per group). The dotted line indicates the average expression level in non-treated (naïve) colonic tissues isolated from TG littermate controls. **Statistics:** Results are pooled from two independent experiments, and data are presented as mean ± SEM, where dots represent individual mice.

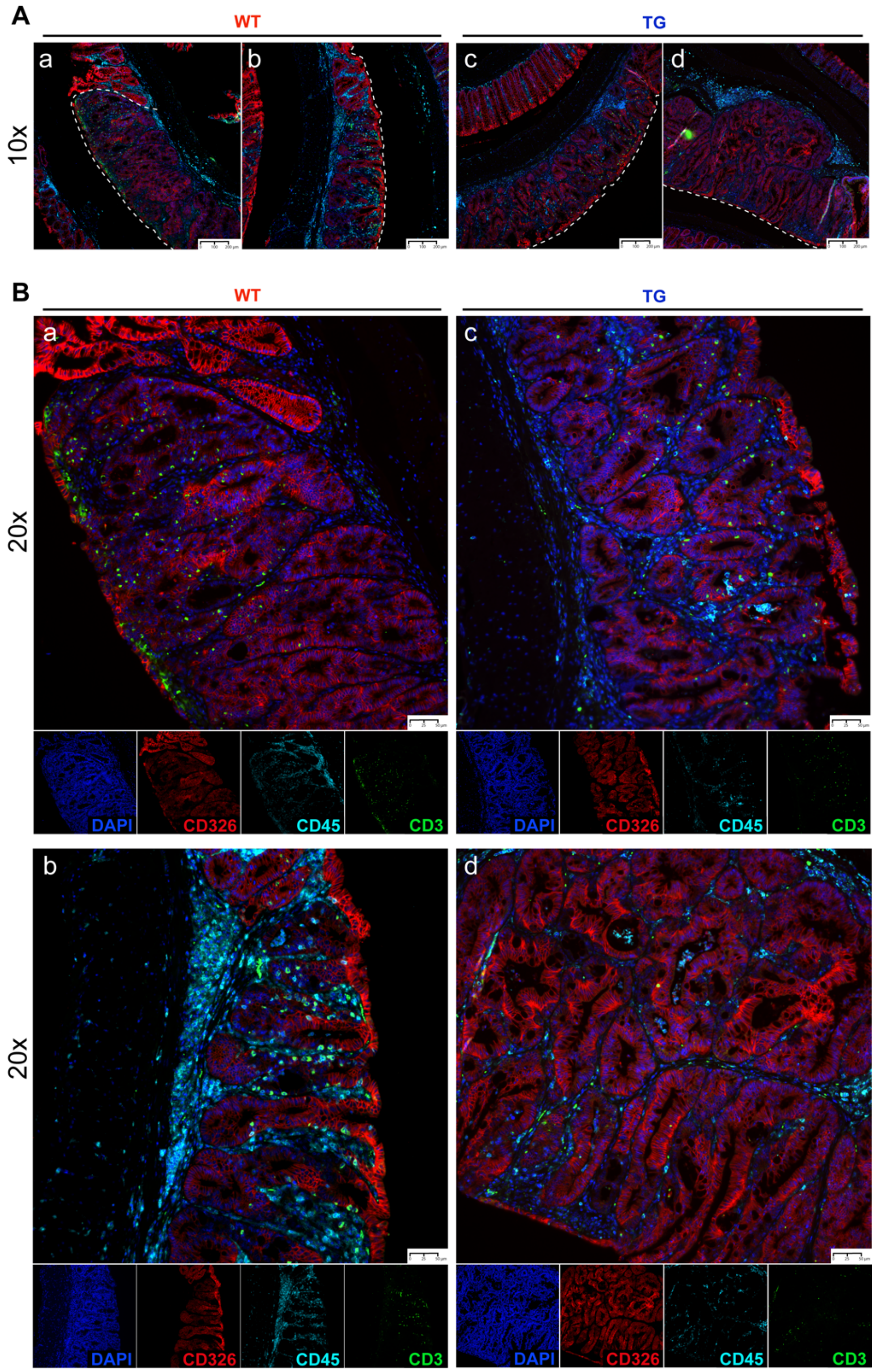

Supplementary Figure 2. Tumor infiltrating immune cells in the mouse model of CAC. (A) Representative immunofluorescence microscopy of well-developed adenocarcinomas from WT and TG littermates. Nuclei (Blue), $CD326^+$ intestinal epithelial cells (Red), $CD45^+$ immune cells (Cyan), and $CD3^+$ T cells (Green) are depicted. The dotted line denotes the luminal surface. **(B)** Representative high-resolution images of the tumors and infiltrating immune cells from WT and TG littermates.

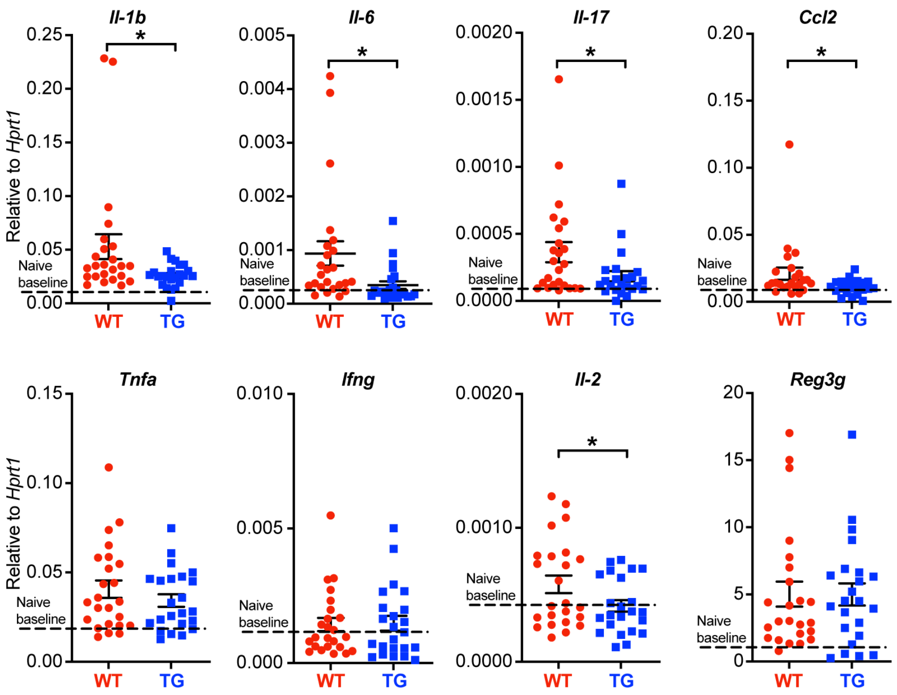

Supplementary Figure 3. FIBCD1 ameliorates the expression of inflammatory markers in non-tumorigenic adjacent tissues. Relative expression of the indicated genes in non-tumorigenic adjacent tissues isolated from WT and TG littermates (n=23-24 mice per group). The dotted line indicates the average level of expression in non-treated colonic tissues isolated from naïve WT and TG littermate controls. **Statistics:** Results are pooled from two independent experiments, and data are presented as mean ± SEM, where dots represent individual mice. Unpaired Student's t test was performed to analyze data. * $p < 0.05$.

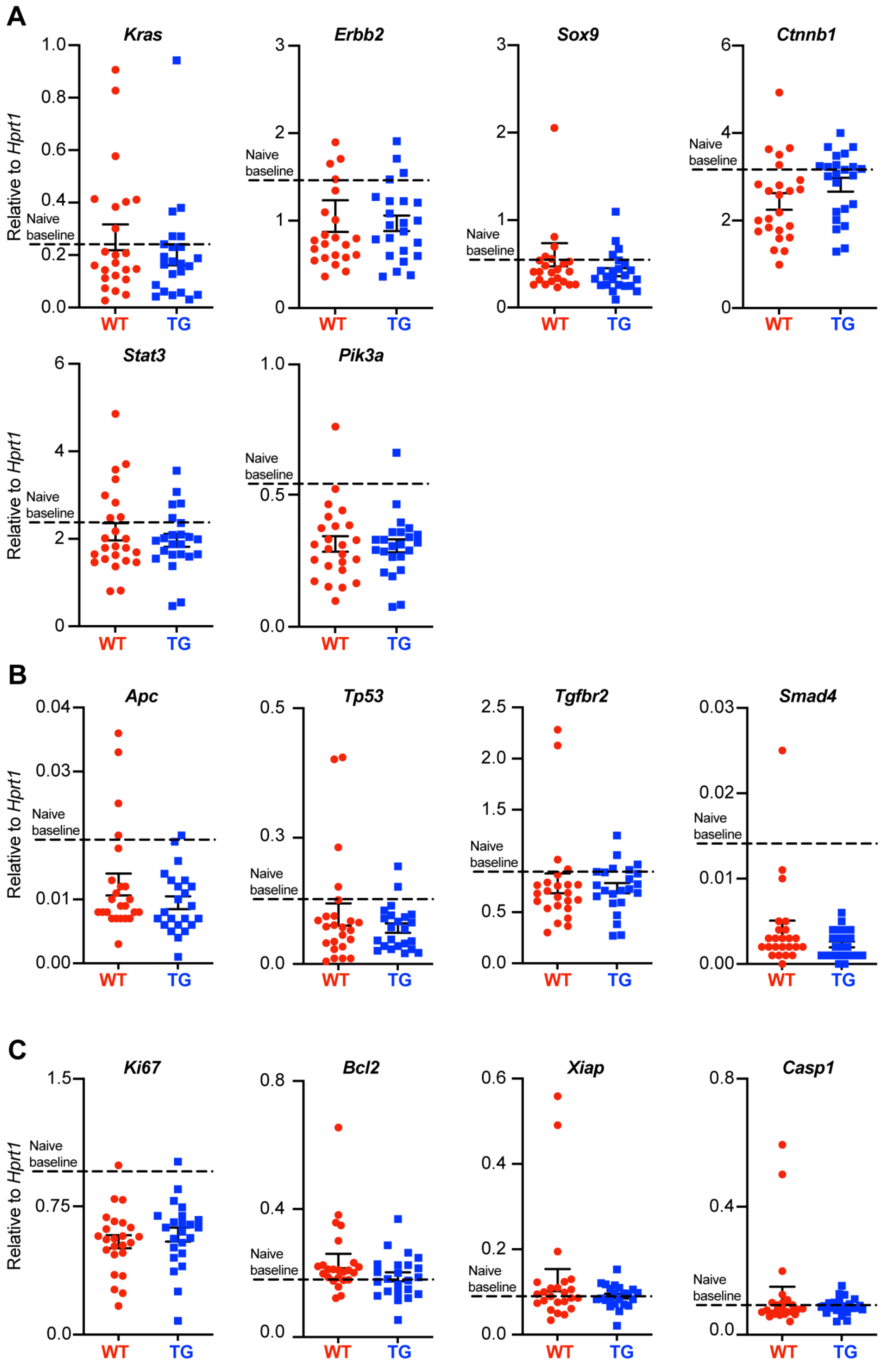

Supplementary Figure 4. FIBCD1 expression does not influence genes related to CAC in non-tumorigenic adjacent tissues. (A) Relative expression of the indicated oncogenes, **(B)** tumor suppressor genes, and **(C)** genes associated with cell proliferation and apoptosis in non-tumorigenic adjacent tissues isolated from WT and TG littermates (n=23-24 mice per group). The dotted line indicates the average level of expression in non-treated colonic tissues isolated from naïve WT and TG littermate controls. **Statistics:** Results are pooled from two independent experiments and data are presented as mean ± SEM where dots represent individual mice. No significant differences were observed when Unpaired Student's t-test was performed to analyze data in (A, B, and C).

Supplementary Table I. Patient characteristics and FIBCD1 immunoscore.

Age	Gender	Cancer classification T	N	M	Stage	FIBCD1 Distribution (0-4)	FIBCD1 Intensity (0-3)	FIBCD1 Immunoscore (0-7)
56	Female	T1	N0	M0	I	0	0	**0**
51	Female	T2	N0	M0	I	0	0	**0**
74	Male	T2	N0	M0	I	0	0	**0**
80	Female	T2	N0	M0	I	0	0	**0**
63	Male	T2	NX	M0	I	1	1	**2**
53	Male	T2	N0	M0	I	0	0	**0**
52	Male	T2	N0	M0	I	4	3	**7**
51	Male	T2	N0	M0	I	2	2	**4**
55	Male	T3	N0	M0	IIA	3	1	**4**
67	Female	T2	N0	M0	IIA	2	3	**5**
79	Male	T3	N0	M0	IIA	2	1	**3**
65	Female	T3	N0	M0	IIA	2	1	**3**
77	Female	T3	N0	M0	IIA	0	0	**0**
47	Female	T3	N0	M0	IIA	2	1	**3**
81	Male	T3	N0	M0	IIA	3	2	**5**
65	Female	T3	N0	M0	IIA	0	0	**0**
82	Male	T3	N0	M0	IIA	1	1	**2**
46	Male	T3	N0	M0	IIA	2	1	**3**
60	Male	T3	N0	M0	IIA	2	1	**3**
64	Male	T3	N0	M0	IIA	3	2	**5**
72	Female	T3	N0	M0	IIA	3	3	**6**
48	Male	T3	N0	M0	IIA	2	1	**3**
74	Female	T3	N0	M0	IIA	4	3	**7**
47	Male	T3	N1a	M0	IIA	2	3	**5**
59	Male	T4a	N0	M0	IIB	0	0	**0**
59	Male	T4a	N0	M0	IIB	2	2	**4**
58	Male	T4a	N0	M0	IIB	1	1	**2**
35	Male	T3	N1a	M0	IIIA	1	1	**2**
56	Male	T2	N1a	M0	IIIA	3	2	**5**
64	Female	T3	N1c	M0	IIIB	1	1	**2**
50	Female	T3	N1a	M0	IIIB	3	1	**4**
82	Male	T3	N1a	M0	IIIB	2	1	**3**
37	Male	T3	N1a	M0	IIIB	4	2	**6**
51	Female	T3	N1b	M0	IIIB	3	3	**6**
66	Female	T4a	N2a	M0	IIIB	4	3	**7**

T=extend of primary tumor, N=extend of regional lymph node involvement, M=Metastasis.
Four patients were excluded from the analysis as carcinoma was not detected in the examined sections
